# Eave tubes for malaria control in Africa: initial development and semi-field evaluations in Tanzania

**DOI:** 10.1186/s12936-016-1499-8

**Published:** 2016-09-01

**Authors:** Eleanore D. Sternberg, Kija R. Ng’habi, Issa N. Lyimo, Stella T. Kessy, Marit Farenhorst, Matthew B. Thomas, Bart G. J. Knols, Ladslaus L. Mnyone

**Affiliations:** 1 Department of Entomology, Center for Infectious Disease Dynamics, the Pennsylvania State University, University Park, USA; 2 Environmental Health and Ecological Sciences Thematic Group, Ifakara Health Institute, P.O.BOX 53, Off Mlabani, Ifakara, Tanzania; 3 In2Care BV, Marijkeweg 22, 6709 PG Wageningen, The Netherlands

**Keywords:** Eave tubes, Semi-field system, House improvement, *Beauveria bassiana*

## Abstract

**Background:**

Presented here are a series of preliminary experiments evaluating “eave tubes”—a technology that combines house screening with a novel method of delivering insecticides for control of malaria mosquitoes.

**Methods:**

Eave tubes were first evaluated with overnight release and recapture of mosquitoes in a screened compartment containing a hut and human sleeper. Recapture numbers were used as a proxy for overnight survival. These trials tested physical characteristics of the eave tubes (height, diameter, angle), and different active ingredients (bendiocarb, LLIN material, fungus). Eave tubes in a hut with closed eaves were also compared to an LLIN protecting a sleeper in a hut with open eaves. Eave tubes were then evaluated in a larger compartment containing a self-replicating mosquito population, vegetation, and multiple houses and cattle sheds. In this “model village”, LLINs were introduced first, followed by eave tubes and associated house modifications.

**Results:**

Initial testing suggested that tubes placed horizontally and at eave height had the biggest impact on mosquito recapture relative to respective controls. Comparison of active ingredients suggested roughly equivalent effects from bendiocarb, LLIN material, and fungal spores (although speed of kill was slower for fungus). The impact of treated netting on recapture rates ranged from 50 to 70 % reduction relative to controls. In subsequent experiments comparing bendiocarb-treated netting in eave tubes against a standard LLIN, the effect size was smaller but the eave tubes with closed eaves performed at least as well as the LLIN with open eaves. In the model village, introducing LLINs led to an approximate 60 % reduction in larval densities and 85 % reduction in indoor catches of host-seeking mosquitoes relative to pre-intervention values. Installing eave tubes and screening further reduced larval density (93 % relative to pre intervention values) and virtually eliminated indoor host-seeking mosquitoes. When the eave tubes and screening were removed, larval and adult catches recovered to pre-eave tube levels.

**Conclusions:**

These trials suggest that the “eave tube” package can impact overnight survival of host-seeking mosquitoes and can suppress mosquito populations, even in a complex environment. Further testing is now required to evaluate the robustness of these findings and demonstrate impact under field conditions.

## Background

Control efforts in sub-Saharan Africa over the past 15 years have prevented an estimated 663 million clinical cases of malaria caused by *Plasmodium falciparum* [1]. Vector control, either in the form of insecticide-treated nets (ITNs) or indoor residual spraying (IRS), is estimated to be responsible for 78 % of those averted cases [1]. In spite of these successes, new interventions are required to boost control of mosquitoes that are not being controlled by existing interventions (e.g. insecticide resistant mosquitoes or outdoor biting mosquitoes), and to provide improved options for insecticide resistance management strategies [2]. The current study presents results of initial investigations into a new intervention called the ‘eave tube’, which aims to address these challenges.

The eave tube technology (which is introduced in [3]) exploits the natural behavioural ecology of the mosquitoes that transmit malaria in sub-Saharan Africa. These mosquitoes have a strong preference for entering homes through the gaps between the walls and the roof—i.e. the eaves [4–7]. Closing off the eaves of houses (together with additional screening of windows) provides a physical barrier that protects inhabitants from malaria [8–11]. It is the physical blocking of mosquito entry into the house that is the major benefit of house improvements in controlling malaria [9, 11, 12]. By reopening small sections of the eaves and installing eave tubes in the openings, mosquitoes are drawn in by the same heat and odour cues that originally attracted them through open eaves. Once inside an eave tube, mosquitoes make contact with insecticide-treated netting placed inside the tube. Thus, in addition to providing a physical barrier to house entry, eave tubes also provide a mosquito killing effect—essentially turning the house into a “lure and kill” device. This effect could potentially suppress mosquito populations or alter population age structures and consequently, achieve community level benefits when coverage is sufficiently high.

Here the development of the eave tube concept in a semi-field system in Tanzania is presented, from initial pilot testing and optimization using overnight release-recaptures through to introducing eave tubes in a six house ‘model village’ with a self-replicating, free living malaria mosquito population and human volunteers and cattle as blood sources.

## Methods

### Overnight release-recapture (experiments 1 and 2)

The mosquitoes used for the overnight release-recapture experiments were *Anopheles arabiensis* from a colony maintained at the Ifakara Health Institute (IHI), originally derived from local mosquitoes collected at a nearby village (Sagamaganga), Tanzania and maintained in this setting for several years. The colony was held in a room within a semi-field screened structure under ambient temperature and relative humidity as described previously described [13]. Larvae were maintained on ground fish meal, adults were provided with sugar water (a 10 % glucose solution), and human volunteers provided blood meals for caged adult female mosquitoes. All experiments used adult female mosquitoes between three and 7 days post-emergence that had not yet received a blood meal. Sugar water was removed from the holding cages of experimental mosquitoes 6 h prior to release in the semi-field compartments.

**Fig. 1:**
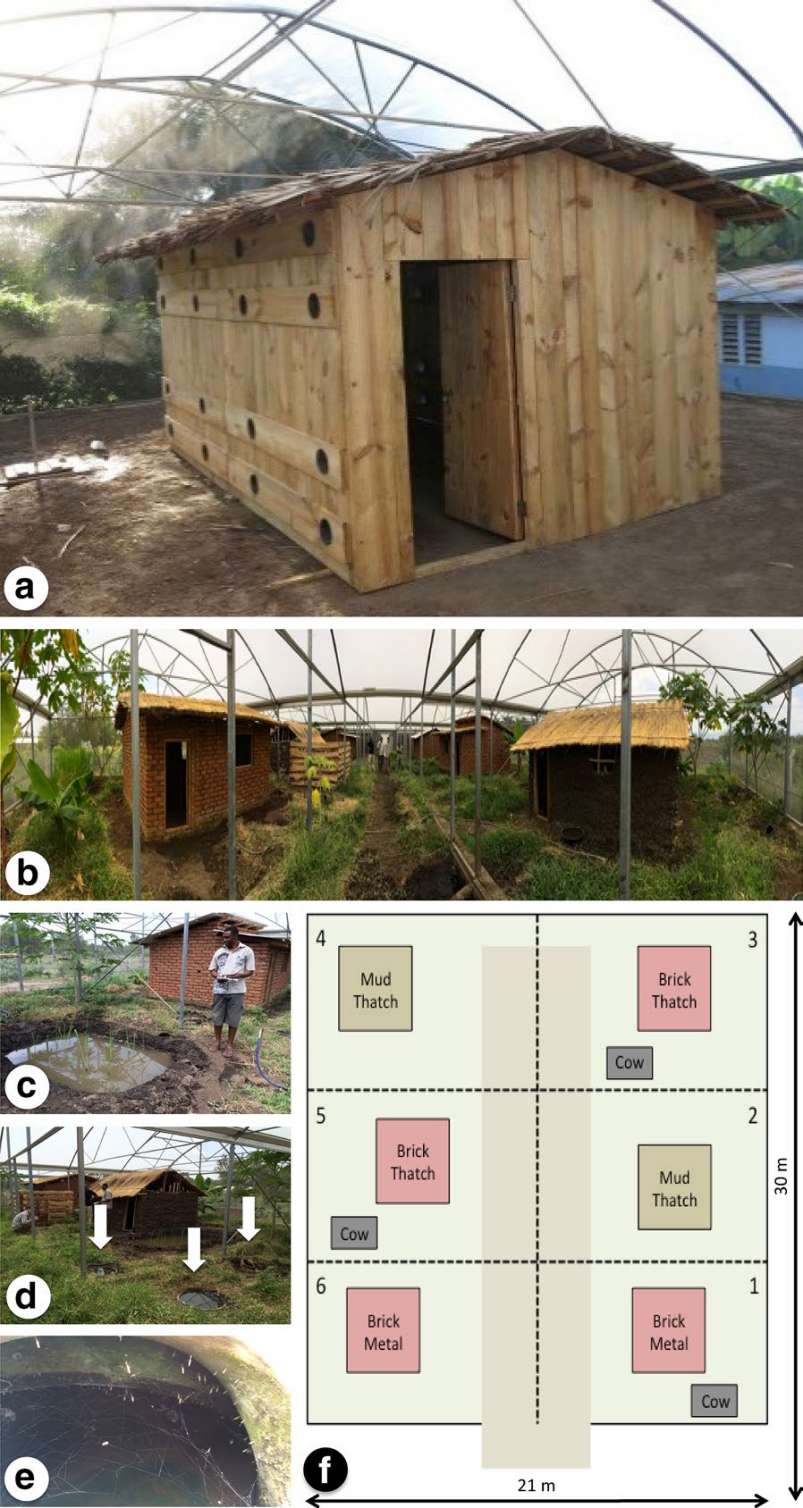
**a** Experimental hut used for initial testing of the eave tube prototype. In this picture, the hut has been modified for experiment 1b (testing eave tubes at different heights). The thatch roof was later replaced with metal sheeting (not pictured). **b** Overview of the semi-field model village showing the six houses. **c** Rice paddy to mimic common breeding sites for *An. arabiensis*. **d** Breeding sites (*left arrows*) and clay pot resting site (*right arrow*). **e** Close-up of resting mosquitoes inside a clay pot. **f** Diagram of model village showing the type and location of houses, cattle sheds, central walkway, and the zones (indicated with *dashed lines*) used for larval sampling. Each zone contained 8–9 larval habitats (51 total)

Inside of a 10 × 10 × 4 m semi-field screened compartment, an experimental hut (4.2 × 2.6 × 2.5 m) was constructed out of wood with a thatch roof (Fig. 1a), later replaced with a metal roof (for experiment 2). This hut did not have any windows or other openings besides the eave tubes and door. The eaves were sealed with wood panels and eight tubes were installed, four on either long side of the hut. As an initial prototype, the tubes in these experiments were pieces of locally available 15.24 cm (6 in.) diameter polyvinyl chloride (PVC) pipes with netting held in place using either a rubber band or a hard plastic ring. At 19:00, shortly after sunset, a human volunteer entered the hut and 200 female mosquitoes were released outside the hut, 50 in. each corner of the compartment. The human volunteer slept under an untreated bed net, unless otherwise indicated.

At 05:00 the next morning, all mosquitoes (both inside the experimental hut and outside in the screened compartment) were recaptured over the course of an hour by two technicians using mouth aspirators. Overnight survival has previously been used as a mosquito fitness measure in a similar screened compartment system [13], and it is a standard measure when testing vector control interventions in experimental huts [14, 15]. However, because of dirt floors in the screened compartments used for this set of experiments, it was difficult to find dead mosquitoes the following morning. Therefore, these experiments used the number of live mosquitoes recaptured in the morning, relative to the number of mosquitoes released the night before, as a proxy for overnight survival.

Recaptured mosquitoes were held in paper cups, with access to sugar water, and their survival was monitored for at least 24 h. Because entomopathogenic fungus causes delayed mortality, survival was monitored up to 3 weeks in the experiment where fungus was used in the eave tubes.

### Experiment 1: pilot testing and optimization

The first series of experiments consisted of simple proof-of-concept trials using overnight release-recapture tests to evaluate the impact of tube size, tube height, tube angle, and choice of insecticide using the simplified hut in Fig. 1a. These experiments are also outlined in Table 1.

**Table 1:** Summary of initial development experiments (experiments 1 and 2) for eave tubes, using the experimental house pictured in Fig. 1a

Experiment	Question	Treatment(s)	Replicates	Measured
1a	How many mosquitoes will pass through open tubes over the course of a night?	1. Open tubes	3 nights total	Number of mosquitoes recaptured inside the house (out of 200 released)
1b	How effective are eave tubes placed at different heights?	1. 20 cm above the ground, treated with bendiocarb 2. 20 cm above the ground, untreated 3. 50 cm above the ground, treated with bendiocarb 4. 50 cm above the ground, untreated 5. 150 cm above the ground, treated with bendiocarb 6. 150 cm above the ground, untreated 7. 180 cm above the ground, treated with bendiocarb 8. 180 cm above the ground, untreated	3 nights per treatment (24 nights total)	Total number of mosquitoes recaptured (out of 200 released)
1c	How effective are eave tubes with different diameters?	1. 10.16 cm in diameter tubes, treated with bendiocarb 2. 15.24 cm in diameter, treated with bendiocarb 3. 15.24 cm in diameter, untreated	3 nights per treatment (9 nights total)	Total number of mosquitoes recaptured (out of 200 released)
1d	Does changing the angle of the tube impact the number of mosquitoes entering through the tubes?	1. Tubes placed at a horizontal angle 2. Tubes placed at an upward angle (highest end inside of the house) 3. Tubes placed at a downward angle (lowest end inside of the house) Note: all treatments tested simultaneously using traps placed on the end of the tubes	9 nights total	Total number of mosquitoes captured inside the eave tube traps (out of 200 released)
1e	How effective are different bioactives in eave tubes?	1. Tubes screened with LLIN material 2. Tubes screened with untreated material 3. Tubes screened with bendiocarb-treated netting, wet formulation 4. Tubes screened with untreated material 5. Tubes screened with bendiocarb-treated netting, dry formulation 6. Tubes screened with untreated material 7. Tubes screened with entomopathogenic fungus (*Beauveria bassiana*). 8. Tubes screened with untreated material Note: each comparison was pairwise between insecticide treatment and untreated material	Treatments 1–6: 3 nights per treatment (18 nights total) Treatments 7 and 8: 2 nights per treatment (4 nights total)	Treatments 1–6: total number of mosquitoes recaptured (out of 200 released). Treatments 7 and 8: proportion surviving (subsample of 50 recaptured mosquitoes)
2a	How does bendiocarb-treated material compare with LLIN material in eave tubes?	1. Eave tubes screened with bendiocarb-treated netting 2. Eave tubes screened with LLIN material 3. Eave tubes screened with untreated netting	7 nights per treatment (21 nights total)	Total number of mosquitoes recaptured (out of 200 released)
2b	How does bendiocarb-treated material in eave tubes compare to an LLIN used (with open eaves)?	1. Eave tubes screen with bendiocarb-treated netting 2. Open eaves, sleeper protected by an LLIN 3. Open eaves, sleeper protected by an untreated net	4 nights per treatment (12 nights total)	Total number of mosquitoes recaptured (out of 200 released)

Eaves were closed and huts had no entry points for mosquitoes, unless otherwise noted. Each experiment used a human volunteer sleeping within the house for all treatments. Note that the word “effective”, as used in the wording of the question, depends on the measured outcome and respective controls

Experiment 1a measured the number of mosquitoes entering the hut when the eave tubes were left unscreened and a volunteer slept inside the hut under an untreated bed net. Experiment 1b measured the number of mosquitoes recaptured when eave tubes were installed at different heights relative to the ground [20, 50, 150 or 180 cm (eave height)] and screened with bendiocarb-treated netting (12 mg/ml of 80 % bendiocarb wettable powder, Ficam W, Bayer AG, Leverkusen, Germany). The height of the eave tubes was changed nightly for twelve nights, with three replicate nights for each height. Recapture of mosquitoes relative to the number released (200) was compared across nights to determine the height at which eave tubes had the greatest impact on recapture and thus presumably, the most contact with mosquitoes attempting to enter the hut.

Experiment 1c measured the mean number of mosquitoes recaptured when 10.16 cm (4 in.) or 15.24 cm (6 in.) eave tubes were installed at eave height. This experiment found no differences between tubes with different diameters, thus 15.24 cm tubes were used for all subsequent experiments.

Experiment 1d tested the impact on catches when tubes were installed at three different angles: upward (approximately 30° relative to the horizontal with the higher end of the tube inside the house), downward (approximately 30° relative to the horizontal with the lower end of the tube inside the house), and horizontal (both ends at the same level). These different orientations were tested simultaneously by using eave tube traps (Sperling et al., pers. comm.) to catch and kill mosquitoes passing through the tubes. These traps were made of a square metal frame covered with bendiocarb-treated netting (12 mg/ml of 80 % bendiocarb wettable powder) and fitted on the indoor end of the eave tubes, without netting in the tube so that mosquitoes could pass into the traps. A total of six tubes were installed in the experimental hut, three on each long side of the house. Each of the three tubes was placed at a different orientation (horizontal, higher end inside the hut, lower end inside the hut), such that each orientation was represented in duplicate (once on either side of the hut). This set up was used to allow a direct comparison between tube orientation and position within a single night. The orientation of each tube was changed nightly for a total of nine nights.

Experiment 1e tested the effect of biological and chemical insecticides on recapture numbers, relative to control nights with untreated netting. These comparisons were: A. Bendiocarb-impregnated netting (12 mg/ml of 80 % bendiocarb wettable powder) versus untreated netting, B. Bendiocarb-dusted electrostatic netting [16] versus untreated netting, C. PermaNet 2.0 (55 mg deltamethrin m^−2^) versus untreated netting, and D. Electrostatic netting dusted to saturation with a 1:1 co-formulation of *Beauveria bassiana* spores and silica (6 g m^−2^) versus untreated netting.

### Experiment 2: comparison of eave tubes and LLINs

Experiment 2 was also conducted using the experimental hut from Fig. 1a and is outlined in Table 1. If eave tubes were not installed, the eaves of the hut were left open.

Experiment 2a compared three types of netting placed within the eave tubes: electrostatic netting coated with powdered bendiocarb (1.25 %, Ficam D, Bayer AG, Leverkusen, Germany), pieces cut from an LLIN (PermaNet 2.0), and untreated electrostatic netting (control). All of the netting was cut into circles with a diameter of 25 cm. The bendiocarb-coated netting was prepared by shaking the circles of netting in a plastic container with 0.24 g of the bendiocarb powder formulation for each piece of netting. The netting was placed on the tubes in the evening before the start of the experiment, and removed the next morning. Tubes were wiped down in between to remove any residue. Prior to the overnight trials, insecticidal activity was confirmed using the MCD bottle bioassay (bioassay method is described in [17]). All three treatments were replicated over seven blocks, for a total of 21 nights. The order of the three treatments within each block was randomized.

Experiment 2b compared a hut with closed eaves and eave tubes treated with bendiocarb (as in experiment 1a) against a hut with open eaves and the sleeper protected either by an LLIN (PermaNet 2.0), or an untreated bed net (control group). The objective of this experiment was to compare the eave tube treatment (including closed eaves) to LLINs (with open eaves). This was a follow up of experiment 2a, where LLIN material was used within eave tubes.

Wood panels fitted with eave tubes (see Fig. 1a) were placed in the open eaves of the experimental hut on the eave tube treatment nights. The panel was removed for the LLIN or control (untreated bed net) nights. The bendiocarb-treated material was prepared and fitted onto the eave tubes using the same method as in experiment 2a. Each treatment was replicated over four blocks for a total of 12 nights. The order of the three treatments within each block was randomized.

### Experiment 3: testing eave tubes in a model village

Experiment 3 tested the impact of eave tubes on a self-replicating mosquito population over time. A simulated village ecosystem was constructed in a 30 × 21 m screened structure compartment (equivalent to six of the single compartments used for overnight release-recapture experiments plus a central walkway; see Fig. 1). Six huts were built in local styles: two traditional houses with mud walls and a grass thatch roof (3 × 4 × 2.5 m), two with brick walls and a corrugated metal sheet roof (3 × 4 × 2.5 m), and two with brick walls and a grass thatch roof (3 × 4 × 2.5 m). In addition to the human dwellings, three cattle sheds were built. Each night, one human volunteer slept in each of the six available houses and two calves were kept in each of the three cattle sheds.

To mimic the surrounding natural ecosystem, vegetation was allowed to grow from seeds present in the soil that was brought into the system. Plants were watered regularly to maintain growth. Fifty-one larval habitats (plastic basins partly filled with soil and tap water, topped off with more water every 2–3 days) and 48 resting places (clay pots, which also served as additional larval habitats) were placed throughout the enclosure. In December 2014, approximately 1200 *Anopheles* larvae were collected from puddles and rice fields near Sagamaganga village and released into the larval habitats inside the model village.

The mosquito population was allowed to grow and stabilize over 4 months, from January to April 2015, after which interventions were introduced in two phases: at the end of April 2015, LLINs were introduced in the four brick huts. Because the two mud huts did not receive LLINs, this resulted in an LLIN coverage of 67 %. At the end of June 2015, all six huts were modified with physical barriers to render them mosquito proof. Windows in all six houses were screened with locally available untreated netting and eave tubes with bendiocarb-treated (1.25 %, Ficam D, Bayer AG, Leverkusen, Germany) electrostatically-charged netting were installed in the four brick huts. The two mud huts received untreated eave screening. After 3 months, in September 2015, the eave tubes (including the filled in eaves), the eave screening (in the mud walled houses), and the window screenings were removed, leaving only bed nets in the model village for the last 4 months of the experiment.

Mosquito populations were monitored every 1–2 weeks by human landing catches (HLC) inside the huts from 19:00 to 01:00, and sampling from larval habitats. During the HLC, six human volunteers were rotated between the six huts to avoid bias in the catches for any given house. The village was sectioned into six zones (8–9 larval habitats per zone) and larval habitats (approximately 3 l containers of water) in the zone were sampled once per sampling time point using a larval dipper (350 ml cups).

### Statistical analysis

In experiments 1 and 2, the number of recaptured mosquitoes was recorded relative to the number of mosquitoes released. In experiment 2, indoor mosquito recapture was recorded, in addition to total recapture (relative to 200 mosquitoes released). To assess the impact of the interventions on recapture in experiment 1, generalized linear models (GLMs) with quasibinomial error distributions were fitted to the data with recapture numbers, relative to the release number, as the outcome, and intervention type included as an explanatory variable. This model was compared to a null model without intervention type. A Turkey all pair comparison was run on the final model using the multcomp package in R (v.3.2.1). To assess the impact of fungus on longer-term survival, a Cox’s proportional hazard model was used with treatment, replicate, and the interaction between treatment and replicate included in the model. Treatment was included in the model as a fixed effect and replicate was included as a random effect. For recapture numbers in experiment 2a, generalized linear mixed effect models (GLMMs) with binomial error distributions were fitted to the data using the lme4 package in R. The type of intervention was included as a fixed effect, and experimental block (from block 1 through 8) was included as a random effect. Likelihood ratio tests were used to compare models with and without the interventions in the model. Odds ratios (OR) and 95 % confidence intervals (CIs) were calculated from estimates and standard errors produced for the full models. For experiment 2b, because there were half as many blocks as in 2a (4 versus 8 blocks), mixed effect models were not used and the analysis was similar to experiment 1; GLMs with quasibinomial error distributions were fitted to the data with recapture numbers (total or indoor only) as the outcome, and intervention type as an explanatory variable.

## Results

### Experiment 1: pilot experiments and optimization

**Fig. 2:**
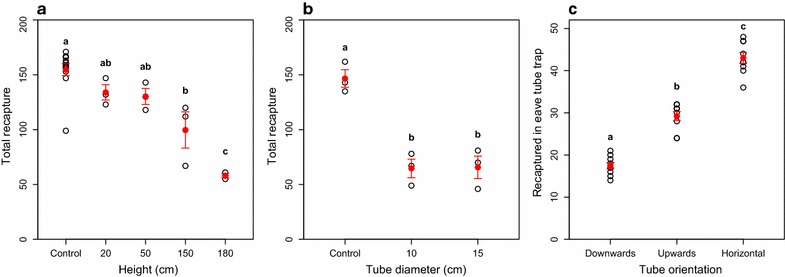
Testing of different physical characteristics of the eave tube. **a** Height of the tubes from the ground, **b** diameter of the eave tubes, and **c** angle of the eave tube, relative to the end inside of the hut (note that because of the use of traps, this is the only experiment where higher numbers of mosquitoes are indicative of mosquitoes contacting the eave tubes). *Open black circles* indicate nightly recapture and *closed red circles* with *error bars* indicate mean recaptures ± SE. *Different letters* indicate significant differences (p < 0.05) based on Tukey all pair comparison

Over three nights, the mean number of mosquitoes entering into the experimental house (Fig. 1a) through open eave tubes was 80.3 ± 4.2 (mean ± SE), or 40 % of the total number (n = 200) released. Placement of eave tubes at eave height (180 cm above the ground) resulted in the highest reduction in recapture relative to the control treatment, where clean netting was placed in the eave tubes (control, mean recapture ± SE: 154.8 ± 5.4; bendiocarb-treated eave tubes placed at 180 cm: 58.0 ± 1.7; Fig. 2a), which is a 62 % reduction in recapture when insecticide-treated netting was used in the eave tubes. This reduction in recapture relative to the control was significant for both eave tubes at 180 and 150 cm (150 cm: t = 4.48, p < 0.001; 180 cm: t = 8.44, p < 0.001) but not for eave tubes at 20 or 50 cm (20 cm: t = 1.59, p = 0.129; 50 cm: t = 1.87, p = 0.076). In tests of 10.16 and 15.24 cm diameter eave tubes, mean recapture was similar for both tube sizes (64.7 ± 8.45 and 65.7 ± 10.3 for 10.16 and 15.24 cm tubes, respectively) and less than half the mean recapture of control nights (146.7 ± 8.0; significant reduction relative to control treatment in 10.16 cm: t = 5.14, p = 0.002, and in 15.24 cm tubes: t = 5.07, p = 0.002; Fig. 2b). In the comparison of the three different eave tube angles (high end of the tube inside the house, low end of the tube inside the house, and both ends at the same level), there was a mean of 91.1 ± 2.7 (mean number captured ± SE) mosquitoes caught nightly inside the eave tube traps, compared to a mean of 76.4 ± 1.5 mosquitoes recaptured outside. Of the mosquitoes caught inside the eave tube traps, there was a significant difference (p < 0.001; Fig. 2c) between all of the tube angles with the most number of mosquitoes found inside the traps attached to horizontal eave tubes (43.0 ± 1.3) compared to tubes slanted upward into the house (29.8 ± 1.3) and tubes slanted downward into the house (18.3 ± 1.03).

**Fig. 3:**
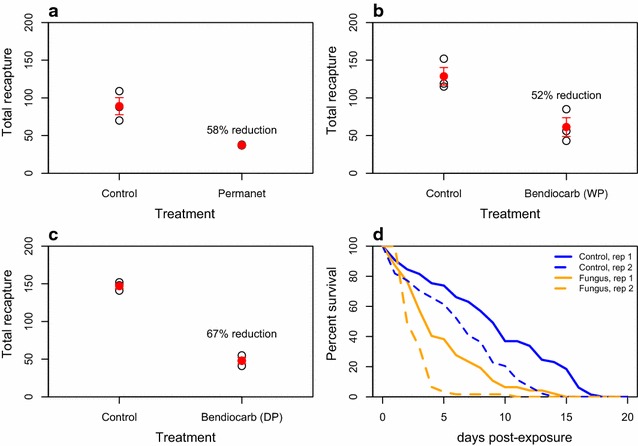
Testing of different bioactives in the eave tubes compared to a control of clean netting; **a** PermaNet (deltamethrin), **b** a wettable powder formulation of bendiocarb (Ficam W), **c** a dry powder formulation of bendiocarb (Ficam D) loaded on electrostatically charged netting, **d** dry fungal spores (*Beauveria bassiana*) loaded on electrostatically charged netting. *Open black circles* indicate nightly recapture and *closed red circles* with *error bars* indicate mean recaptures ± SE. *Lines* in **d** show mean cumulative survival for each day

The number of mosquitoes recaptured was significantly reduced compared to controls for all of the chemical insecticides that were tested (Fig. 3); 58 % for PermaNet 2.0 (t = 5.63, p = 0.005), 52 % for the wettable bendiocarb (Ficam W) treated netting (t = 3.58, p = 0.023), and 67 % for dry bendiocarb-dusted (Ficam D) electrostatic netting (t = 14.5, p = 0.0001). These results demonstrate that eave tubes can potentially kill, within a single night, up to two-thirds of the released mosquitoes. Longer term survival was also significantly reduced in mosquitoes released overnight in compartments where fungus-treated eave tubes had been installed in the experimental hut, compared to the control group where untreated netting was used (hazard ratio = 3.7, p < 0.001). Average survival was 4.3 ± 0.2 days (mean ± SE) in the group exposed to *B. bassiana* compared to 9.1 ± 0.5 days for the control mosquitoes.

### Experiment 2: comparison of eave tubes and LLINs

**Fig. 4:**
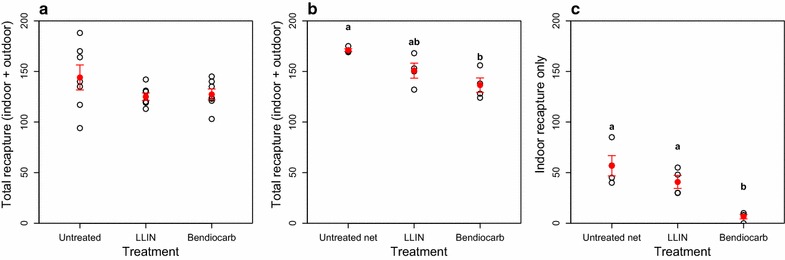
Comparing eave tubes and LLINs. **a** Eave tubes screened either with netting cut from an LLIN (PermaNet 2.0), electrostatic netting treated with bendiocarb powder, or untreated netting (control). **b** Total recapture (inside and outside the experimental house), eaves closed and eave tubes installed with bendiocarb-treated electrostatic netting or eaves open and sleeper protected by an LLIN or an untreated net (control). **c** Indoor only recapture for the same experiment shown in **b**. *Open black circles* indicate nightly recapture and *closed red circles* with *error bars* indicate mean recaptures ± SE. *Different letters* indicate significant differences (p < 0.05) based on Tukey all pair comparison

In experiment 2a, insecticidal netting in the eave tubes had a significant effect on the number of mosquitoes recaptured the following morning (effect of treatment: X^2^ = 6.42, df = 2, p = 0.040) relative to eave tubes with clean netting (control group). In both insecticide treatment groups (LLIN material or bendiocarb-treated material), the odds of recapturing the mosquitoes released the night before were significantly lower (LLIN material: OR = 0.87, 95 % CI (0.77, 0.98), p = 0.021; Bendiocarb treated material: OR = 0.88, 95 % CI (0.79, 0.99), p = 0.042; Fig. 4a) than in the control group. These results indicate that both LLIN material (PermaNet 2.0, treated with deltamethrin) and electrostatic netting treated with bendiocarb powder can reduce overnight survival of *An. arabiensis*, compared to untreated netting. Although some mosquito mortality did occur in the 24 h following recapture, the level of mortality was consistent across treatments. This suggests that, with the insecticides that were tested, the impact of eave tubes is primarily a result of overnight mortality and not delayed mortality.

In experiment 2b, the total number of mosquitoes recaptured in the morning (both inside the experimental house and outside in the screened compartment) was reduced both by the use of an LLIN with open eaves and by closed eaves with bendiocarb-treated eave tubes, relative to the control group (open eaves and untreated bed net), but the effect was significant only in the closed eaves and eave tube group (LLIN: t = 2.22, p = 0.054; eave tubes: t = 3.91, p = 0.004; Fig. 4b). The effect of treatment (open eaves and LLIN, closed eave and eave tubes, or open eaves and untreated bed net) on recapture of mosquitoes inside the experimental hut (“indoor recapture”) was similar and even more pronounced (LLIN: t = 1.48, p = 0.17; eave tubes: t = 5.17, p = 0.001; Fig. 4c). This was not surprising considering that there was both a physical barrier (closed eaves) and insecticide treatment in the eave tube group.

The results from experiment 2 show that, although LLIN material in eave tubes performed as well as bendiocarb-treated material, when the eave tubes are compared to LLIN material used as a bed net, the eave tubes treatment had a significantly greater reduction in mosquito recapture.

### Experiment 3: testing eave tubes in a model village

**Fig. 5:**
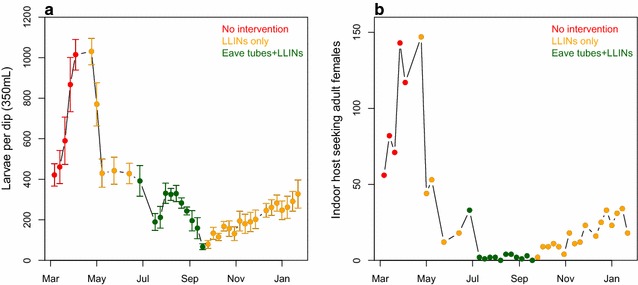
Mosquito recapture numbers in the model village. **a** Larval numbers over time, measured using dippers to sample larval habitats. *Points* and *error bars* indicate the mean number of larvae collected in a larval habitats (±SE) for each sampling time point. **b** Host seeking adult female numbers over time, measured using indoor human landing catches (*HLC*). *Points* indicate the total number of mosquitoes recaptured throughout a night for each sampling time point

Four months after the initial introduction of larvae, in January 2015, the average number of larvae collected in dip samples (350 ml of water) taken in each of the six sampling zones (larval habitats of approximately 3 l) around the enclosure was 1015 ± 75 (mean ± SE) and the total number of host-seeking females collected indoors by a human landing catch over the course of a night was 117. In April 2015, LLINs were introduced into the model village. Two months after the introduction of LLINs, the mean larval catch was 428 ± 47 (42 % of the original catch; Fig. 5a) and total indoor host-seeking female catch was 18 (15 % of the original catch; Fig. 5b). At this point, eave tubes and window screening were installed in four of the six houses in the village. Three months after the introduction of eave tubes, mean larval abundance was further reduced to 67.0 ± 14.5 (7 % of the original catch or 16 % of the final catch prior to the introduction of eave tubes) and no adult females were recaptured indoors. After 3 months, during which time both LLINs and eave tubes were present, the window screens and eave tubes were removed and the eaves were opened again, after which the mosquito population started to gradually recover. In the final sampling at the end of January (13 months after the introduction of mosquitoes into the model village), the mean larval catch had recovered to 328 ± 68.9 and the total indoor adult female catch was 18 (32 and 15 % of the catches prior to the initial introduction of LLINs, for larvae and adult females, respectively).

## Discussion

The concept of eave tubes emerged out of an existing body of knowledge implicating the open eaves of African houses as a primary entry point for malaria mosquitoes [3, 7–9]. The development of eave tubes within screened compartments at the Ifakara Health Institute in Tanzania is presented here. Overnight release-recapture experiments were carried out in similar settings to those previously used, for example, to test the impact of fungal insecticide treatment and host meal source [13, 18]. A self-sustaining mosquito population was also established in a “model village”, based on previous experience with creating such populations at IHI [19–21]. Unlike prior semi-field systems, however, the “model village” included multiple human dwellings occupying a larger space, along with a rice paddy and multiple cattle sheds, to better emulate a Tanzanian village environment. The variability represented in the model village includes different house designs (Fig. 1), from the more traditional mud walls and thatch roofs to the more modern brick walls and metal roofs. The brick walled houses received both eave tubes and associated house screening while the mud walled houses received only the screening. This is because mud walled houses are not amenable for easy installation of eave tubes [3].

The goal of these studies was rapid development of field-ready technology. To meet this goal, development of the eave tube concept began with a series of pilot studies using overnight releases of mosquitoes in a screened compartment with a simplified experimental hut (Fig. 1a). These experiments, outlined in Table 1, served as proof of concept, demonstrating that mosquitoes do indeed pass through eave tubes to enter into a house, and treating eave tubes with insecticides will reduce the number of mosquitoes that are recaptured the following morning. This set of experiments included some optimization of the design and testing of different insecticides.

Eave tubes were also tested in this setting (i.e. a single, simplified wooden experimental hut) with material cut from a commercially available LLIN (PermaNet 2.0) and compared to eave tubes with bendiocarb-treated electrostatically-charged netting (a recently developed technology for improving insecticide bioavailability [16]), together with an untreated control. Both types of insecticide-treated netting worked equally well for reducing mosquito recapture when used in eave tubes (with closed eaves). However, when closed eaves fitted with bendiocarb-treated eave tubes were compared to open eaves with the sleeper protected under an LLIN, recapture was lower in the group of mosquitoes released in the compartment with the bendiocarb-treated eave tubes and closed eaves.

Even with these promising results, some variation in the effect size of eave tubes across the different experiments was observed. This could be due to a number of factors including seasonal variation and differences between volunteer sleepers that made them more or less attractive to mosquitoes, or modifications to the experimental hut where the thatch roof was removed and replaced with sheet metal. Follow up experiments, including refinement of the prototype and testing in other semi-field systems with other species of *Anopheles* (Snetselaar et al., pers. comm.), and filming of mosquito behaviour in eave tubes (Sperling et al., pers. comm.), will be helpful to identify potential sources of variation and improve the technology.

Lastly, both LLINs and bendiocarb-treated eave tubes were introduced into a model village in the screened structure. The model village consisted of six houses and three cattle sheds, with volunteers and cattle brought in overnight to maintain a mosquito population within the screened structure. The introduction of LLINs reduced the abundance of host seeking females within the huts to 15 % and the abundance of larvae to 42 % of the original population, a residual population possibly supported by the presence of non-human hosts (i.e. cattle). Following the introduction of closed eaves and eave tubes treated with bendiocarb in the brick houses, plus screening of open eaves in mud walled houses and screening of windows in all houses, indoor biting mosquitoes were virtually eliminated and the larval population was further reduced to 7 % of its original size. These results represent only a single replicate population (with no control population) over the course of a year, due to time and logistic constraints, and thus it is difficult to account for stochastic variation or seasonal effects, or to directly compare a treatment and control populations. However, the population dynamics are consistent with eave tubes and associated screening offering additional control, on top of what can be obtained with the frontline intervention consisting of LLINs. Interestingly, suppression of the mosquito population occurred despite the presence of unprotected cattle in the model village, which suggests that even with alternative hosts present and a mosquito species known to exhibit zoophilic feeding behaviour, eave tubes can have an effect on a population of anopheline mosquitoes. In other words, these results suggest that even a zoophilic species like *An. arabiensis* still makes sufficiently frequent contact with eave tubes (presumably during attempts to enter human dwellings) to suppress the population. The impact of eave tube technology on the more anthropophilic malaria vector species, such as *Anopheles gambiae* s.s. and *Anopheles funestus*, could be even more pronounced.

Although these results indicate that eave tubes are a technology worth pursuing, numerous questions remain. For example, although eave tubes are a promising delivery mechanism for bioactives or insecticides that are not currently being used for malaria vector control, additional testing is necessary. This includes direct comparisons between insecticides like bendiocarb (currently used for IRS) and deltamethrin (currently used for LLINs and IRS), and a bioactive like entomopathogenic fungus (not currently used for vector control). Similarly, eave tubes should be tested against insecticide resistant mosquitoes.

One potential benefit of eave tubes is the ease of re-treatment, which has clear benefits for cost and logistical constraints, but it also provides the opportunity for resistance management strategies. For example, multiple eave tubes in the same house might be treated with different insecticides, or even loaded with multiple insecticides within the same tube, as a ‘combination therapy’ approach similar to the strategies used to manage drug resistance in malaria parasites. However, extensive theoretical and empirical work is still needed to determine exactly which strategies would be most effective for resistance management.

Another caveat for the experiments presented here is that, in the overnight release-recapture experiments, a wooden house with no windows was used. This is obviously a highly simplified version of a typical home environment, which does not allow for multiple sources of heat and odour cues. More realistic brick or mud-walled houses were later constructed in the model village. Additionally, because eave tubes are a house-based intervention, like LLINs and IRS, they do not necessarily address current pressing concerns regarding outdoor biting or behavioural resistance [22–24]. However, there is evidence that over 80 % of successful feeding events by mosquitoes old enough to transmit malaria will happen after at least one house entry attempt [25], which would preserve the effectiveness of house-based interventions like eave tubes, LLINs, and IRS. Whether eave tubes (and any associated house modifications) are cost-competitive with IRS will require further, detailed economic analyses. Most importantly, large-scale field trials are needed to determine whether the reduced survival and suppression of mosquito populations observed in these semi-field experiments translates into entomological and, ultimately, epidemiological impacts in the real world.

## Conclusions

Overnight trials conducted in a screened compartment containing a single house guided the initial development of the eave tube prototype, and suggest that eave can impact the overnight survival of host-seeking mosquitoes.

Eave tubes, along with associated screening of windows, were then introduced into a large compartment containing a self-sustaining mosquito population, a six house “model village” with volunteer sleepers protected under bed nets, and cattle housed in cattle sheds. The resulting decline in mosquito populations following this introduction suggest that eave tubes and associated screening can suppress mosquito populations and reduce the potential for indoor biting, beyond the impact of LLINs alone and even in a complex environment with alternative hosts present.

## Abbreviations

LLIN: long-lasting insecticidal net
IRS: indoor residual spraying
IHI: Ifakara Health Institute
HLC: human landing catch
MCD: mosquito contamination device
GLM: generalized linear model
GLMM: generalized linear mixed model
SE: standard error
OR: odds ratio
CI: confidence interval
